# Mental Fatigue Has Great Impact on the Fractal Dimension of Brain Functional Network

**DOI:** 10.1155/2020/8825547

**Published:** 2020-11-12

**Authors:** Gang Li, Yanting Xu, Yonghua Jiang, Weidong Jiao, Wanxiu Xu, Jianhua Zhang

**Affiliations:** ^1^Key Laboratory of Urban Rail Transit Intelligent Operation and Maintenance Technology & Equipment of Zhejiang Provincial, Zhejiang Normal University, Zhejiang 321005, China; ^2^College of Engineering, Zhejiang Normal University, Jinhua 321004, China; ^3^Xingzhi College, Zhejiang Normal University, Lanxi 321100, China; ^4^Key Laboratory of High Efficiency and Clean Mechanical Manufacture, Ministry of Education of China, School of Mechanical Engineering, Shandong University, Jinan 250061, China

## Abstract

Mental fatigue has serious negative impacts on the brain cognitive functions and has been widely explored by the means of brain functional networks with the neuroimaging technique of electroencephalogram (EEG). Recently, several researchers reported that brain functional network constructed from EEG signals has fractal feature, raising an important question: what are the effects of mental fatigue on the fractal dimension of brain functional network? In the present study, the EEG data of alpha1 rhythm (8-10 Hz) at task state obtained by a mental fatigue model were chosen to construct brain functional networks. A modified greedy colouring algorithm was proposed for fractal dimension calculation in both binary and weighted brain functional networks. The results indicate that brain functional networks still maintain fractal structures even when the brain is at fatigue state; fractal dimension presented an increasing trend along with the deepening of mental fatigue fractal dimension of the weighted network was more sensitive to mental fatigue than that of binary network. Our current results suggested that mental fatigue has great regular impacts on the fractal dimension in both binary and weighted brain functional networks.

## 1. Introduction

Mental fatigue has become a widespread subhealthy condition in nowadays society [1]. Many researchers are dedicated to the studies of detection methods [2], neural mechanisms [3], and mitigation methods [4] of the mental fatigue, owing to the negative effects of mental fatigue on the human cognitive functions [5], especially serious in driving fatigue [6]. Recently, mental fatigue has been prevalently explored by the means of brain functional network with the neuroimaging technique of electroencephalogram (EEG) [7–9]. It has been widely proved that mental fatigue can lead to distinct changes in brain functional network structures, for example, the changes of small-world property [3, 10]. However, to our knowledge, no researcher has studied the effects of mental fatigue on the fractal feature of brain functional network.

**Figure 1:**
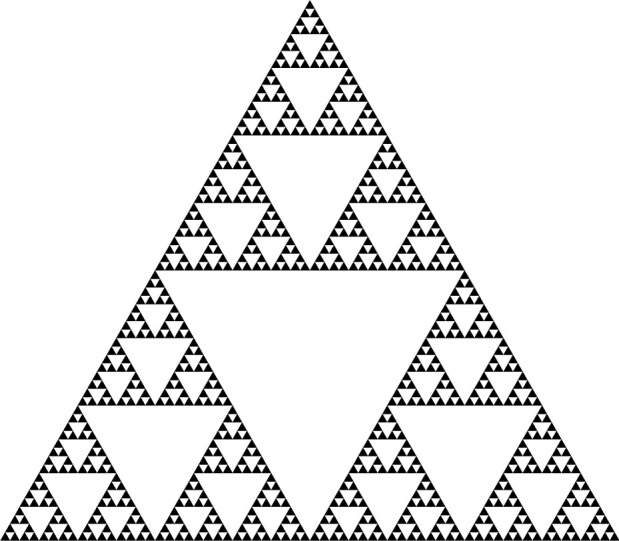
Sierpinski triangle. As shown in the figure, the whole and the part have strict self-similarity.

Fractality was first proposed by the French mathematician Mandelrot in 1967 when he was trying to figure out how to measure the coastline [11]. Mandelbrot defines that a fractal is a shape made of parts similar to the whole in some way, see Figure 1 for the example. Fractal has been found to widely exist in nature [12]. Since the fractal theory was revealed, it has been widely used in biomedical signal analysis, such as EEG [13], fMRI (functional magnetic resonance imaging) [14], and MRI [15]. Naturally, a scientific question arises: does the brain functional network have fractal feature?

Brain functional network, as one type of the complex networks in statistical physics, is a demonstration of the temporal correlations between different brain areas in the processes of neural physiological events [16]. It has become one of the most widely used techniques to investigate the neurodynamics of cognitive functions [17–20], which are especially sensitive to mental fatigue [3, 9]. Since the small-world characteristic [21] and scale-free property [22] of complex networks were proposed, the studies of complex network topology have entered a high-speed developing period [23]. Nevertheless, it was generally believed that complex networks do not have self-similarity at the beginning of the small-world characteristic discovery [24]. In other words, complex networks do not show fractal property. The small-world network requires that the diameter of the complex network slowly increases with the increase of the number of network nodes, and the network nodes and diameter have an exponential relationship, while the self-similar structure requires them to satisfy the power-law relationship [25]. However, Song et al. have revealed that most real networks have self-similar structures by using renormalization method in 2005 [25]. By performing box-covering method on the complex networks [26], they discovered that the box number, referring to the number of boxes covering the entire network for the given box size and ensuring that the diameter of the box is smaller than the given box size, has a power-law relationship with the box sizes, which revealed a common self-similarity of the complex networks. From then on, the fractal property of complex networks attracted much attention among the researchers. It has been proved that brain functional networks have fractal property [27, 28].

In the study of fractal features of complex networks, the self-similarity is easy to be measured and described, and fractal dimension is the direct quantification of the self-similarity of fractal structures [26]. Fractal dimension can be measured and calculated by the box-covering method introduced by Song et al. [26]. When computing the fractal dimension of a complex network, the most important and difficult step is to find the minimum box number with the given box size. Song et al. gave two equivalent algorithms for calculating the minimum box number, greedy colouring algorithm, and burning algorithm [26], but these two algorithms need to repeat the calculation many times when estimating the fractal dimension of the complex networks. Therefore, many researches focus on fractal dimension calculation algorithms in complex networks [27, 29].

**Figure 2:**
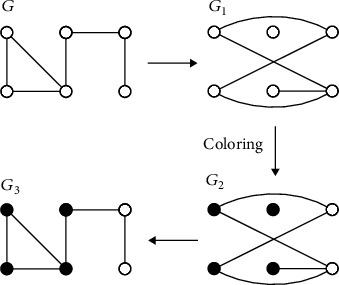
Greedy colouring algorithm flowchart [26].

In this study, the greedy colouring algorithm was chosen and modified to calculate the fractal dimension of brain functional networks during the formation of mental fatigue. The flowchart of greedy colouring algorithm is shown in Figure 2 [26]. For the given network *G* and given box size *L*_*B*_ = 3, connect the nodes that the distance (refer to shortest path length) between the nodes in the network *G* is greater than or equal to 3, resulting in the new network *G*_1_. Then, the network *G*_1_ is coloured by traversing all the network nodes, which should ensure that the two connected nodes are painted with different colours, resulting in the coloured network *G*_2_. After the completion of colouring network *G*_2_, the original connectivity is restored to gain network *G*_3_. The number of colours in network *G*_3_ is the box number covering the entire network *G*_3_ for the given box size *L*_*B*_. Then, fractal dimension *d*_*B*_ can be determined by equation (1) [25]. Greedy colouring algorithm can turn the problem of finding the minimum box number into the problem of colouring all nodes, which is easy to be implemented by computer programming. However, in the first step of the greedy colouring algorithm [26], the network nodes need to be numbered randomly, which may make a big difference for the box number obtained by different network nodes' numbering orders. Therefore, this algorithm introduced by Song et al. needs to repeat the calculation 10000 times to reduce the influence of randomness and improve the accuracy of the fractal dimension calculation. In the current study, we would modify the greedy colouring algorithm to reduce the calculated number of repetitions.

(1) $$\begin{array}{c} N_{B}\approx L_{B}^{-d_{B}}. \end{array}$$

In the current study, we attempted to investigate the effects of mental fatigue on the fractal dimension of the brain functional network with a modified method. For this purpose, firstly, a challenging sustained mental arithmetic math task was performed on twenty young male volunteers for mental fatigue induction, and multichannel EEG data were recorded both in resting state and task state before and after the tasks. Secondly, binary and weighted brain functional networks were constructed with two widely used methods using mutual information to determine the functional connectivity among all pairwise combinations of EEG channels. Finally, the fractal dimensions were calculated based on our improved greedy colouring algorithm both in binary and weighted brain functional networks, which were then used to analyze the impacts of mental fatigue on the brain functional networks.

## 2. Materials and Methods

### 2.1. Participants

Twenty healthy and right-handed male engineering postgraduate students (females and left-handed volunteers were excluded to eliminate the effects of sex differences and left-right handedness differences on the results) were enrolled. They were postgraduate college students, aged 24.5 ± 1.5 years, and their mean BMI (body mass index) was 20.7 ± 1.8kg/m^2^. All subjects were asked to read and sign an informed consent form before the test, and the Shandong University Ethics Committee have approved this study. This experiment complied with the principles set forth in the Helsinki declaration (2013 revision), the international code of ethics for biomedical research involving human beings (2016), the world declaration on the human genome and human rights (1997), and other relevant ethical requirements. Every individual should have a regular life, normal eyesight, and no brain disorders. Each participant was required to not stay up late and not drink alcohol and drugs within one week before the experiment. They were prohibited from smoking and having coffee and tea in 8 hours and demanded to wash their hair in 2 hours before EEG recording. All participants would get some monetary reimbursement to motivate them to cooperate better during the experiment.

### 2.2. EEG Data Acquisition and Preprocessing

**Figure 3:**
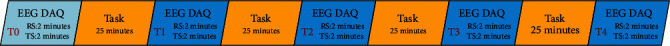
EEG data acquisition (EEG DAQ) procedures. RS means resting state and TS means task state.

Nineteen-channel EEG data were collected by an EEG apparatus (SYMTOP NT9200) according to the international 10-20 system. The sampling rate was 1000 Hz, and the electrode impedance was controlled below 5000 *Ω*. Every participant was required to do a continuous mental arithmetic task with 200 different problems (a double-digit between sixty and ninety plus another random double-digit between sixty and ninety and then multiplied by a random single digit between six and nine). Each mental arithmetic problem was designed to be completed in 30 seconds determined by preceding pretests. That is, all the participants can get high accuracies during the four tasks (the whole task was equally divided into 4 task segments). The results of the accuracies were similar and had no statistical difference among these four tasks. What we are concerned was the effects of continuous tasks on the brain functional network structures. The participants were required to highly concentrate on calculating the given mental arithmetic math problems and write down the answers on a paper. Besides, 2-minute EEG data for resting state and 2-minute EEG data for task state were recorded before and after each task segment, resulting in 5 times EEG data acquisitions named as T0, T1, T2, T3, and T4, see Figure 3 for details. Resting state refers to closing the eyes, being awake, and relaxed. Meanwhile, task state means keeping the body still and doing a mental arithmetic problem, a three-digit subtracts a single-digit continuously. All the mental arithmetic math problems were showed on a computer screen and displayed automatically controlled by a timer. The whole experiment was implemented from 7 pm to 9 pm in a sound-attenuated and light, temperature, and humidity-controlled room.

In the current study, two subjects' EEG data were eliminated on account of their big head movements when recording EEG. EEG data at task state were downsampled from 1000 Hz to 256 Hz. EEG rhythms was extracted by a digital FFT filter (firstly, EEG signal was converted into its frequency spectrum by FFT, and only the interest frequency range is retained; secondly, interest EEG frequency band was obtained by inverse FFT). Ten pieces of five seconds of continuous EEG data for each state were singled out for functional connectivity computation. Here, only alpha1 rhythm (8-10 Hz) at task state, which had significant statistical difference for mutual information in mental fatigue detection, was further analyzed according to our previous study [30].

### 2.3. Brain Functional Network Construction

The mutual information (see reference [31] for detailed definition and description) between all pairs of EEG channels was computed to determine functional connectivity by a software proposed by Moddemeijer [32], obtaining an undirected 19 × 19 adjacency matrix for alpha1 rhythm at task state. Adjacency matrix is a means of representing which nodes of a network are adjacent to which other nodes. Then, two typical methods, named as method I and method II for convenient presentation, were used to convert the adjacency matrixes into brain functional networks. Method I refers to using a certain weight of the functional connectivity to delete the network edges whose weights are smaller than the selected weight. Whereas method II means keeping the number of network edges fixed in the networks by selecting the edges from high to low according to the weights. The main difference between these two methods is that method I probably makes the number of network edges inconsistent among the obtained networks, while method II makes all obtained networks have the same number of network edges. Method I can sufficiently consider the effects of weights on the network structure. If the whole weights in an adjacency matrix are higher than those in another one, the converted network can contain more network edges, which would directly result in bigger differences of the network features between these networks. As for method II, all obtained networks have the same number of nodes and edges, and the only difference is in the spatial arrangement of network edges. Then, we can compare the topological structures to distinguish network features between different networks. In this study, both binary and weighted brain functional networks obtained with these two methods were taken into consideration. Binary brain functional network means setting the weights of the edges to 1, whereas weighted brain functional network refers to keeping the weights changeless.

### 2.4. Modified Algorithm for Fractal Dimension Computation

In order to reduce the repetitive computation times and improve the efficiency of fractal dimension calculation, we propose to arrange the node degrees (node degree means the number of edges connected to the node) in descending order during the colouring step based on the Welch-Powell algorithm [33]. In other words, the nodes are coloured in the order of node degree from high to low. Meanwhile, the nodes with the same degree are randomized to eliminate the effect of the order of the same node degree on the result of fractal dimension calculation when colouring. The specific algorithm steps for binary brain functional network are as follows (see Figure 2 for an example):

**Step 1 (a).**

Calculate the shortest path length *l*_*ij*_ (see reference [30] for detailed description and definition) between all nodes in network *G*.

**Step 2 (b).**

Set the box size *L*_*B*_ = 1.

**Step 3 (c).**

Connect the nodes between node *i* and node *j* when *l*_*ij*_ ≥ *L*_*B*_ in network *G* to get the new network *G*_1_.

**Step 4 (d).**

Calculate all node degrees of network *G*_1_, add a small random noise less than 1 on the node degrees to randomize the same node degrees, and then arrange all the nodes in descending order based on the node degrees to get the sequential numbering from 1 to *n* for every node. Put node 1 and other nodes which are not connected to it into the first box; put the smallest node numbering in the remaining nodes and other nodes which are not connected to it into the other box until all nodes are processed. The total number of used boxes is *N*_*B*_ for the given *L*_*B*_.

**Step 5 (e).**

Increase *L*_*B*_ by one, repeat step (c) and step (d) until *L*_*B*_ = *L*max*B*(*L*max*B* refers to the maximum value of *l*_*ij*_).

**Step 6 (f).**

Linear fit log(*L*_*B*_) and log(*N*_*B*_), and take the absolute value of its slope as the fractal dimension *d*_*B*_.

Basing on the principle of constructing box size [29] and the idea of node sequencing in the improved algorithm proposed above, the specific algorithm steps for weighted brain functional network are determined as follows (see Figure 2 for an example):

**Step 7 (a).**

Calculate the weighted shortest path length *lwij* (see reference [30] for detailed description and definition) between all nodes in network *G*, and arrange all weighted shortest path length values in ascending order as *d*1, *d*2, *d*3, •••, *dk*, •••, and*dn*.

**Step 8 (b).**

Set box size *L*_*B*_ = *d*k, and *k* = 1.

**Step 9 (c).**

Connect the nodes between node *i* and node *j* when *lwij* ≥ *L*_*B*_ in network *G* to get the new network *G*_1_, which is resulted as a binary network.

**Step 10 (d).**

Calculate all node degrees of network *G*_1_, add a small random noise less than 1 on the node degrees to randomize the same node degrees, and then arrange all the nodes in descending order based on the node degrees to get the sequential numbering from 1 to *n* for every node. Put node 1 and other nodes which are not connected to it into the first box; put the smallest node numbering in the remaining nodes and other nodes which are not connected to it into the other box until all nodes are processed. The total number of used boxes is *N*_*B*_ for the given *L*_*B*_.

**Step 11 (e).**

Increase *k* by one, meanwhile increase *L*_*B*_ by *d*k, repeat step (c) and step (d) until *L*_*B*_ ≥ *LmaxB* = *d*n.

**Step 12 (f).**

Linear fit log(*L*_*B*_) and log(*N*_*B*_), and take the absolute value of its slope as the fractal dimension *d*_*B*_.

Additionally, a group of thresholds, 0.2, 0.25, and 0.3 for method I and 45, 70, and 90 for method II, were selected to construct both binary and weighted brain functional networks with method I and method II, respectively. The thresholds for method I and method II should be neither too big nor too small, which must ensure that no isolated nodes existed in the networks, and the structural differences were enlarged among T0, T1, T2, T3, and T4. A total of 100 repetitions were performed under each threshold, and all the results of fractal dimension *d*_*B*_ were averaged. The least-square method is adopted for the linear fitting of log(*L*_*B*_) and log(*N*_*B*_) to obtain the fractal dimension *d*_*B*_ of binary and weighted brain functional networks using cftool Toolbox embedded in MATLAB 2012b.

### 2.5. Statistical Analysis

One-way analysis of variance (ANOVA) was carried out for the fractal dimensions to distinguish the statistically significant differences among T0, T1, T2, T3, and T4. *P* value is given as the ANOVA results. Results are displayed as mean ± SD (standard deviation). Significant level is reported at *P* < 0.01.

## 3. Results

**Figure 4:**
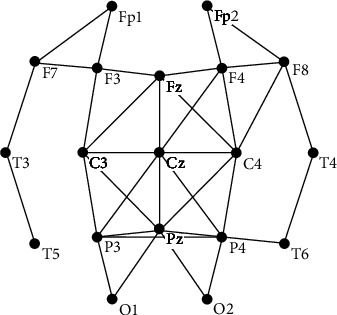
An example of brain functional network structure gained with method I for alpha1 rhythm at task state in T0 time period.

Figure 4 shows an example of the brain functional network. Tables 1–3 are obtained from 10 random computations on the same brain functional network given in Figure 4.  Table 1 is the calculated box numbers using the traditional greedy colouring algorithm. As shown in Table 1, the results of the box numbers are quite different among the 10 groups obtained by different numbering sequence, and this phenomenon is pretty significant under the box size of 4, 5, and 6. Meanwhile, this traditional algorithm performs poorly to obtain the minimum box number under the box size of 2 and 3. Tables 2 and 3 are the results of box numbers obtained by the modified greedy colouring algorithm in binary and weighted networks, respectively. In order to clarify that the modified greedy colouring algorithm is better than the traditional greedy colouring algorithm, we computed the adjusted *R*-square (which can be used as the evaluation index of fitting quality) of linear fitting between log(*L*_*B*_) and log(*N*_*B*_). The results of adjusted *R*-square corresponding to Tables 1–3 are shown in Table 4. As shown in Table 4, modified greedy colouring algorithm can obtain 0.9563 ± 0.0024 and 0.9450 ± 0.0131 for binary and weighted networks, respectively, whereas traditional algorithm can only obtain 0.3541 ± 0.1043 for binary networks. As shown in Tables 1–4, the modified algorithm can effectively improve the accuracy of calculating the box number and can gain a better approximation of the box number under the limited computations. Different ordering of nodes with the same degree in network *G*_1_ (see Figure 2) have an effect on the results of the box numbers, which can be eliminated by repeating calculations with the improved algorithm.

**Figure 5:**
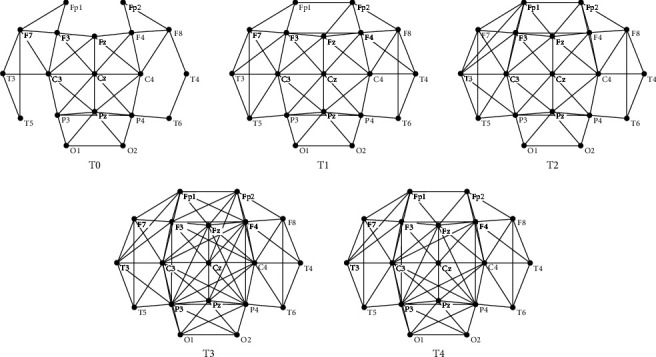
Brain functional network structures during the formation of mental fatigue obtained by the average of all participants.

**Figure 6:**
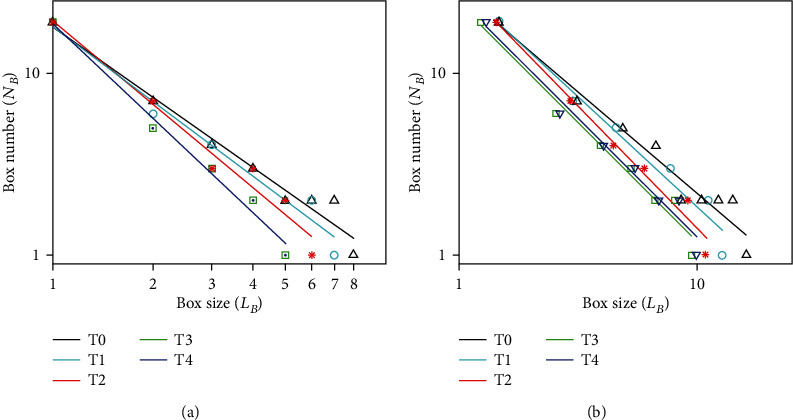
The relationship between box size *L*_*B*_ and box number *N*_*B*_ in binary and weighted brain functional networks during the formation of mental fatigue. (a) Results in binary brain functional network. (b) Results in weighted brain functional network.

Figure 5 demonstrates a group of brain functional networks obtained by method I during the formation of mental fatigue.  Figure 6 is the linear fitting results between log(*L*_*B*_) and log(*N*_*B*_) for T0, T1, T2, T3, and T4 in both binary and weighted brain functional networks on the basis of Figure 5.  Table 5 is the results of fit quality corresponding to Figure 6. The values shown in Table 5 are all above 0.94, which shows a good linearity to prove that both binary and weighted brain functional networks have fractal feature. The box sizes corresponding to Figure 6 are counted as shown in Table 6. Table 6 shows that the fractal dimensions of T3 and T4 networks can be distinguished by the difference in box sizes in weighted networks, but there is no difference in the binary network. The results demonstrate that the number of box sizes needed in weighted network is more than that in binary network, which suggested a better box coverage in the weighted brain functional network than in the binary brain functional network.

**Figure 7:**
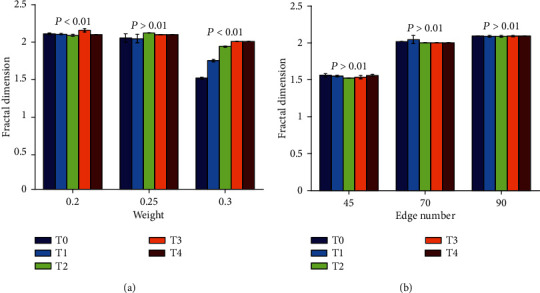
Results of fractal dimension obtained by method I and method II in binary brain functional network during the formation of mental fatigue. The bars indicate the standard error of mean. (a) Results in method I. (b) Results in method II.

**Figure 8:**
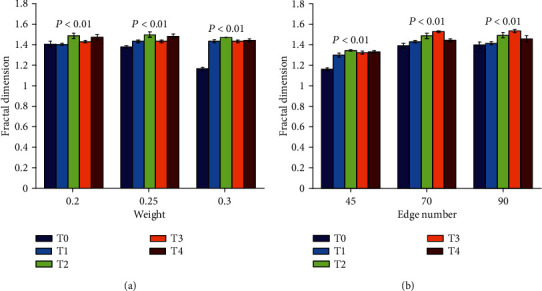
Results of fractal dimension obtained by method I and method II in weighted brain functional network during the formation of mental fatigue. The bars indicate the standard error of mean. (a) Results in method I. (b) Results in method II.

Figures 7 and 8 are the results of fractal dimensions under different thresholds computed by the improved greedy colouring algorithm in binary and weighted brain functional network under method I and method II, respectively. As shown in Figure 7(a), it is observed that the fractal dimension of binary brain functional networks increases significantly with the accumulation of task time only when the threshold values are 0.2 and 0.3 in method I (*P* < 0.01). In method II shown in Figure 7(b), there is no significant change in the fractal dimension of five time periods under all given thresholds. As shown in Figure 8, the fractal dimension of weighted brain functional networks in both methods I and II shows a significant increasing trend (*P* < 0.01). By comparing Figures 8(a) and 8(b), it can be found that method II is better in depicting fractal dimension than method I, and its variation trend is more consistent.

## 4. Discussion

In this study, we explored the effects of mental fatigue on the fractal dimension in both binary and weighted brain functional networks with the EEG data of alpha1 rhythm at task state. Box-covering method of greedy colouring algorithm was used to calculate the fractal dimension of the brain functional network, which was known to belong to the NP-hard problems for the minimum box number identification [26]. Song et al. had to repeat the calculation 10000 times to acquire the fractal dimension with greedy colouring algorithm [26]. So the greedy colouring algorithm needed to be improved to raise the computing efficiency. Comparing Tables 2 and 3 with Table 1, the results indicated that the modified greedy colouring algorithm can effectively obtain the minimum box number for the given box size and can solve the problem of colouring the nodes that have the same degree by adding a small random noise on the node degrees during renormalization process. According to Table 4, this improved algorithm is better than the traditional algorithm, which can be used to investigate the fractal dimension of the brain functional network during the formation of mental fatigue.

In order to determine whether the brain functional network had fractal characteristic during the formation of mental fatigue, a group of brain functional networks at T0, T1, T2, T3, and T4 were chosen to compute the fractal dimension. The results shown in Figure 6 and Table 4 indicated that both the binary and weighted brain functional networks still maintained fractal structure even when the brain was at fatigue state. Moreover, the fractal dimension of T3 and T4 in the binary brain functional network cannot be distinguished, but it can be identified in the weighted network, which initially reflected the advantage of the fractal dimension in weighted brain functional network. The number of box sizes in the weighted network was also slightly larger than that in the binary network, which suggested the more refined box coverage in the weighted brain functional network. Therefore, we can conclude that the fractal dimension of the weighted brain functional network was indeed better than that of the binary network in response to the change of brain functional state.

The fractal dimension demonstrated an increasing trend along with the accumulation of task time in both binary and weighted brain functional networks. Fractal dimension was an effective indicator to measure the complexity and irregularity of self-similar structures [27, 34]. The results indicated that mental fatigue can lead to a higher complexity of brain functional network structure, and the brain's ability to maintain more complex functional networks in task state may further lead to the deepening of mental fatigue. In the binary brain functional network (see the results of Figure 7), the fractal dimension tended to increase as the level of mental fatigue increases in method I and has no significant changes in method II, which reflected that the response of method I to mental fatigue is slightly better than method II. In the weighted brain functional network (see the results of Figure 8), the weights had a greater influence on the fractal dimension than the number of network edges, because the changing trend of fractal dimension under method II (in which the number of network edges was the same among T0, T1, T2, T3, and T4) was more consistent than that under method I. Therefore, it can be concluded from the comparison between binary and weighted networks that the fractal dimension of the weighted brain functional network was more sensitive to mental fatigue than that of the binary network.

Previous studies have proved that the small-world characteristic and fractal characteristic of brain functional networks can indeed coexist [35–37]. When analyzing the origin of fractal structure in the evolution process of complex networks [24], Song et al. pointed out that the mutual exclusion (or mismatches) between the central nodes of complex networks can cause the central nodes to disperse with each other, which generated the fractal characteristic of the complex networks. In the brain functional network, the center nodes are not completely exclusive, but there is certain connectivity between the center nodes, which probably makes the characteristic path length between any two nodes become shorter, exhibiting small-world characteristic in the network. That is to say, the coexistence of small-world feature and fractal feature in the brain functional networks may be caused by neither complete attraction nor complete exclusion between central nodes. Previous researchers have reported that the small-world feature still exists in the brain functional networks when the brain is at fatigue state [3, 10, 38]. Taking together with the results of this study, we can infer that the small-world feature and fractal feature of the brain functional networks can still exist simultaneously during the formation of mental fatigue.

## 5. Conclusions

In the present study, we focused on studying the effects of mental fatigue on the fractal dimension in brain functional networks with a modified greedy colouring algorithm. The results suggested the following conclusions: first, the improved greedy colouring algorithm can efficiently obtain the minimum box number for the given box size, which can reduce the repetitive computation times and improve the efficiency of fractal dimension calculation; second, both binary and weighted brain functional networks still maintain fractal structure even when the brain is at fatigue state, and the fractal dimension presents an increasing trend along with the accumulation of task time, which indicated that the topological structures of brain functional networks become more complex with the increasing of mental fatigue; third, the fractal dimension of the weighted brain functional network is indeed better than that of the binary network in response to the change of brain functional state; finally, it is of great reason to infer that the small-world feature and fractal feature of the brain functional networks can still exist simultaneously during the formation of mental fatigue. Our results provide a better method for fractal dimension estimation and a new perspective to understand the neural mechanisms of mental fatigue based on the fractal feature of the complex networks.

## Figures and Tables

**Table 1:** The box numbers *N*_*B*_ under the box sizes *L*_*B*_ acquired by greedy colouring algorithm in a binary brain functional network.

Box size (*L*_*B*_)	1	2	3	4	5	6	7	8
Box number (*N*_*B*_)	19	18	18	17	15	9	3	1
	19	19	19	19	11	4	2	1
	19	19	19	12	7	4	3	1
	19	19	19	18	16	9	3	1
	19	19	17	14	11	8	3	1
	19	19	19	19	16	9	3	1
	19	19	19	19	15	6	3	1
	19	19	19	17	14	9	3	1
	19	19	19	19	15	8	3	1
	19	19	17	14	9	4	2	1

**Table 2:** The box numbers *N*_*B*_ under the box sizes *L*_*B*_ acquired by improved greedy colouring algorithm in a binary brain functional network.

Box size (*L*_*B*_)	1	2	3	4	5	6	7	8
Box number (*N*_*B*_)	19	7	4	3	2	2	2	1
	19	8	4	3	2	2	2	1
	19	7	4	3	2	2	2	1
	19	7	5	3	2	2	2	1
	19	7	5	3	2	2	2	1
	19	8	4	3	2	2	2	1
	19	8	5	3	2	2	2	1
	19	8	5	3	2	2	2	1
	19	8	5	3	2	2	2	1
	19	7	4	3	2	2	2	1

**Table 3:** The box numbers *N*_*B*_ under the box sizes *L*_*B*_ acquired by improved greedy colouring algorithm in weighted brain functional network.

Box size (*L*_*B*_)	1.47	3.14	4.88	6.69	8.55	10.41	12.27	14.14	16.02
Box number (*N*_*B*_)	19	7	5	4	2	2	2	2	1
	19	8	5	4	2	2	2	2	1
	19	7	5	4	3	2	2	2	1
	19	7	5	4	2	2	2	2	1
	19	7	5	4	2	2	2	2	1
	19	8	5	4	2	2	2	2	1
	19	8	5	4	2	2	2	2	1
	19	8	5	4	2	2	2	2	1
	19	8	5	4	3	2	2	2	1
	19	7	5	4	3	2	2	2	1

**Table 4:** The results of fitting quality (adjusted *R*-square) for traditional method and modified method in binary and weighted networks according to the results of Tables 1–3.

	Traditional algorithm	Modified algorithm in binary network	Modified algorithm in weighted network
1	0.2691	0.9576	0.9338
2	0.4024	0.9594	0.9417
3	0.5621	0.9576	0.9522
4	0.2505	0.9529	0.9338
5	0.3690	0.9529	0.9338
6	0.2612	0.9594	0.9417
7	0.3533	0.9553	0.9417
8	0.2805	0.9553	0.9417
9	0.3058	0.9553	0.9771
10	0.4871	0.9576	0.9522
Mean ± SD	0.3541 ± 0.1043	0.9563 ± 0.0024	0.9450 ± 0.0131

**Table 5:** The results of fitting quality (adjusted *R*-square) for T0, T1, T2, T3, and T4 in binary and weighted brain functional networks corresponding to Figure 6.

Time	T0	T1	T2	T3	T4
Binary network	0.9638	0.9659	0.9556	0.9818	0.9818
Weighted network	0.9476	0.9583	0.9741	0.9665	0.9655

**Table 6:** The box size LB used in the improved greedy colouring algorithm in binary and weighted brain functional networks corresponding to Figure 6.

Network type	Time	Box size (*L*_*B*_)								
Binary network	T0	1	2	3	4	5	6	7	8	—
	T1	1	2	3	4	5	6	7	—	—
	T2	1	2	3	4	5	6	—	—	—
	T3	1	2	3	4	5	—	—	—	—
	T4	1	2	3	4	5	—	—	—	—

Weighted network	T0	1.47	3.14	4.88	6.69	8.55	10.41	12.27	14.14	16.02
	T1	1.46	3.01	4.59	6.17	7.78	9.41	11.05	12.70	—
	T2	1.44	2.92	4.45	5.99	7.58	9.18	10.83	—	—
	T3	1.23	2.56	3.91	5.28	6.64	8.02	9.42	—	—
	T4	1.30	2.65	4.07	5.50	6.95	8.44	9.93	—	—

## Data Availability

The data used to support the findings of this study are available from the corresponding author upon request.

## Abbreviations

EEG: Electroencephalogram
fMRI: Functional magnetic resonance imaging
BMI: Body mass index.
